# Exploring stroke discourse on Twitter through content and network analysis among Indian users

**DOI:** 10.1038/s41598-024-65858-9

**Published:** 2024-07-02

**Authors:** Thilagavathi Ramamoorthy, Vaitheeswaran Kulothungan, Bagavandas Mappillairaju

**Affiliations:** 1 School of Public Health, SRM Institute of Science and Technology, Kattankulathur, Chengalpattu District, Tamil Nadu 603 203 India; 2 ICMR-National Centre for Disease Informatics and Research, Bengaluru, Karnataka 562110 India; 3 Centre for Statistics, SRM Institute of Science and Technology, Kattankulathur, Chengalpattu District, Tamil Nadu 603 203 India

**Keywords:** Stroke, Social media, India, Social network analysis, Unsupervised machine learning, Health care, Mathematics and computing

## Abstract

The study aimed to understand stroke-related Twitter conversations in India, focusing on topics, message sources, reach, and influential users to provide insights to stakeholders regarding community needs for knowledge, support, and interventions. Geo-tagged Twitter posts focusing on stroke originating from India and, spanning from November 7, 2022, to February 28, 2023, were systematically obtained via the Twitter application programming interface, using keywords and hashtags sourced through Symplur Signals. Preprocessing involved the removal of hashtags, stop words, and URLs. The Latent Dirichlet Allocation (LDA) topic model was used to identify recurring stroke-related topics, while influential users were identified through social network analysis. About half of the tweets about stroke in India were about seeking support and post-stroke bereavement sharing and had the highest reachability. Four out of 10 tweets were from the individual twitter users. Tweets on the topic risk factors, awareness and prevention (14.6%) constituted the least proportion, whereas the topic management, research, and promotion had the least retweet ratio. Twitter demonstrates significant potential as a platform for both disseminating and acquiring stroke-related information within the Indian context. The identified topics and understanding of the content of discussion offer valuable resources to public health professionals and organizations to develop targeted educational and engagement strategies for the relevant audience.

## Introduction

Stroke is a major global health concern, with significant economic and health consequences. The World Health Organization (WHO) reports that stroke ranks as the second leading cause of global mortality and the third major contributor to disability-adjusted life years (DALYs). The burden of stroke has been increasing over time, with a substantial rise in the absolute number of cases from 1990 to 2019, especially in low and middle income countries^1^. Stroke is responsible for 7.4% of all deaths and 3.7% of all disability-adjusted life years (DALYs) in India in 2019, with 0.45% annual percent change in death indicating increasing burden^2^. This increase has been driven by population growth, ageing, and changes in risk factors such as high systolic blood pressure, diabetes, high body mass index, smoking, and unhealthy diets. This highlights the urgent need for effective primary prevention, early diagnosis and treatment strategies, particularly in regions where resources may be limited.

Social media, including Twitter, has revolutionized the way healthcare professionals, health advocates, patients and care providers communicate and disseminate information on prevention and treatment^3^. Twitter, recently renamed as “X” with its succinct format, allows for quick and easy sharing and seeking of ideas, news, and research findings and become an important platform for knowledge-sharing, networking, and patient engagement. Stakeholders like healthcare organisations, physicians and other healthcare professionals used Twitter to share their research findings, medical news, create awareness and updates on the risk factors, symptoms, latest treatments and procedures for various health conditions^4–7^. Health advocates used Twitter to promote public health campaigns, raise awareness about various diseases and health conditions, and advocate for policies that support better health outcomes. They also used Twitter to connect with patients and their families, answer questions, and share information about available resources and support groups^8,9^. Patients used Twitter to connect with other patients who have similar health conditions, share their experiences and provide support for one another. Patients also used Twitter to find healthcare providers, ask questions about their conditions, and stay up-to-date on the latest treatments and research^10^.

Globally, discussions related to stroke in social media has been used to understand the conversations on heat waves, quality and trustworthiness of information, treatment, recovery, and survivorship^11,12^. The usage of twitter and other social media in India for health information and communication has been previously studied for Polycystic ovary syndrome, diabetes, cancer and COVID-19 in context of vaccine as well as its psychological impacts^13–17^. A study conducted in the Indian context examined the marketing approaches employed by e-cigarette marketers on social media, as well as the level of user engagement. The study revealed that social media plays a significant role in communication, and marketers employed customized marketing techniques to ensure effective communication^18^.

The field of stroke medicine is in a constant state of flux, as it swiftly adapts to integrate the latest therapies and guidelines^19^. It has been observed that social media serves as a valuable ally in medical education, facilitating communication between patients and doctors, raising awareness, and providing a platform for individuals to share their opinions^20^. The key to effective stroke diagnosis is to act "FAST", and generating awareness among the general public should involve addressing the specific needs of the target population. Exploring the use of Twitter for stroke-related communication can provide valuable insights into the current status, content, and needs of relevant stakeholders in India. The objective of this study was to analyse the stroke-related discussions on Twitter in India by extracting the topics of conversation and identifying the key influencers using content and network analysis. The study's objective was to address the following research questions:

1. What are the topics of discussion in Twitter regarding stroke in India?
2. What is the extent of reachability and engagement of the stroke related Twitter messages?
3. Who are the primary influencers behind the stroke-related discussions on Twitter in India, and what are their characteristics?

## Methods

### Data collection

Twitter is a widely-used social media platform for sharing small pieces of information on a global scale, and it serves as a valuable source of data for conducting content and network analyses. The keywords pertaining to stroke were chosen from the Symplur Healthcare Hashtag Project, which compiles a range of hashtags associated with different medical conditions and health issues. The keywords include ‘stroke’, ‘stroke awareness’, ‘stroke prevention’, and ‘India’. In addition, manual searching was also conducted to identify stroke-related content specific to India on Twitter. Tweets from November 7th, 2022, to February 28th, 2023, were acquired using Twitter's REST Application Programming Interface (API) with the assistance of the R package "rtweet". We gathered publicly accessible messages that included at least one of the stroke-related keywords, along with associated user information. The collected tweets were consolidated and duplicates were removed by examining the text and the user's screen name. We excluded tweets geolocated outside of India and tweets not in the English language. Pre-processing of tweets to remove non-english terms, stop words, URLs, links and stemming was performed.

### Topic modeling and evaluation

Topic models are statistical techniques that discern pivotal words or phrases encapsulating the content within a document, facilitating the categorization of similar documents. Latent Dirichlet Allocation (LDA), a widely used unsupervised machine learning technique for topic modeling^21^, has been employed extensively to identify hidden topics associated with various health conditions and diseases^22,23^. In this study, LDA was applied using Python libraries like spaCy and gensim, with a lambda parameter (λ) ranging from 0.6 to 0.9 and 3000 Gibbs sampling iterations. The optimal number of topics was determined using coherence scores (Multimedia Appendix 1), learning decay plot(Multimedia Appendix 2) and perplexity. A coherence score measures topic similarity, while perplexity assesses prediction accuracy. Four topics were selected based on these metrics. LDAvis, a Python tool, was used to visualize these topics and identify the top 10 words for each, which helped assign concise names to each theme. The intertopic distance map (Multimedia Appendix 3) and the 30 most relevant terms were also presented visually. Further analysis involved using t-distributed stochastic neighbor embedding to visualize document clusters (Multimedia Appendix 4). Finally, 20% of tweets in each topic were manually evaluated to validate and refine topic titles and understand public perception.

### Network analysis

Network analysis was performed to identify the influential nodes who are important in spreading information related to stroke and related risk factors. Network metric measures such as degree, betweeness centrality, eigen vector were calculated. Gephi software was used for this analysis and visualization.

### Source and reachability evaluation

The tweet sources were examined and manually categorized into the following groups: healthcare organizations, individuals, health related individuals, doctors, media and others including non-health organizations. We assessed tweet reachability by considering the number of favorites(likes) and retweets(shares). These metrics indicate the tweets' popularity and depict user interactions, with higher counts signifying greater reach. We calculated topic-specific and user-type-based reachability by comparing the retweet or favorite count to the total number of tweets within the respective topic or user category.

### Ethical approval

The study was approved by the institutional ethics review committee from SRM. Medical college hospital and research centre—SRM Institute of Science and Technology (Approval no: 2989/IEC/2021).

## Results

A total of 5179 stroke related tweets from 3381 users were retrieved for the period 7^th^ November 2022 and 28^th^ February 2023. The tweet distribution throughout the study period can be found in Multimedia Appendix 5. Among these tweets, 68.8% were unique tweets, 55.1% had mentions, 26.8% had website links and 24.5% had media objects (photos/videos). (Table 1) The volume of tweets were slightly higher during December and January (56.6%) than compared to the remaining study period. Those users identified as individuals generated the most stroke related tweets (52.4%), followed by doctors (16.3%), media (12.5%) and healthcare organisations like hospitals, health product companies, pharma companies and medical associations (11.8%). (Multimedia Appendix 6).

**Table 1:** Summary of key users and tweet descriptors of Stroke Discourse on Twitter in India (November 2022 - February 2023).

Data descriptor	Total count
Unique users	3381
Tweets generated	5179
Tweets with unique messages	3561
Tweets with mentions	2853
Tweets that were retweets	1385
Tweets with links	1389
Tweets with media (photo, video)	1270
Tweets that were replies	1377

### Topic modelling

**Figure 1:**
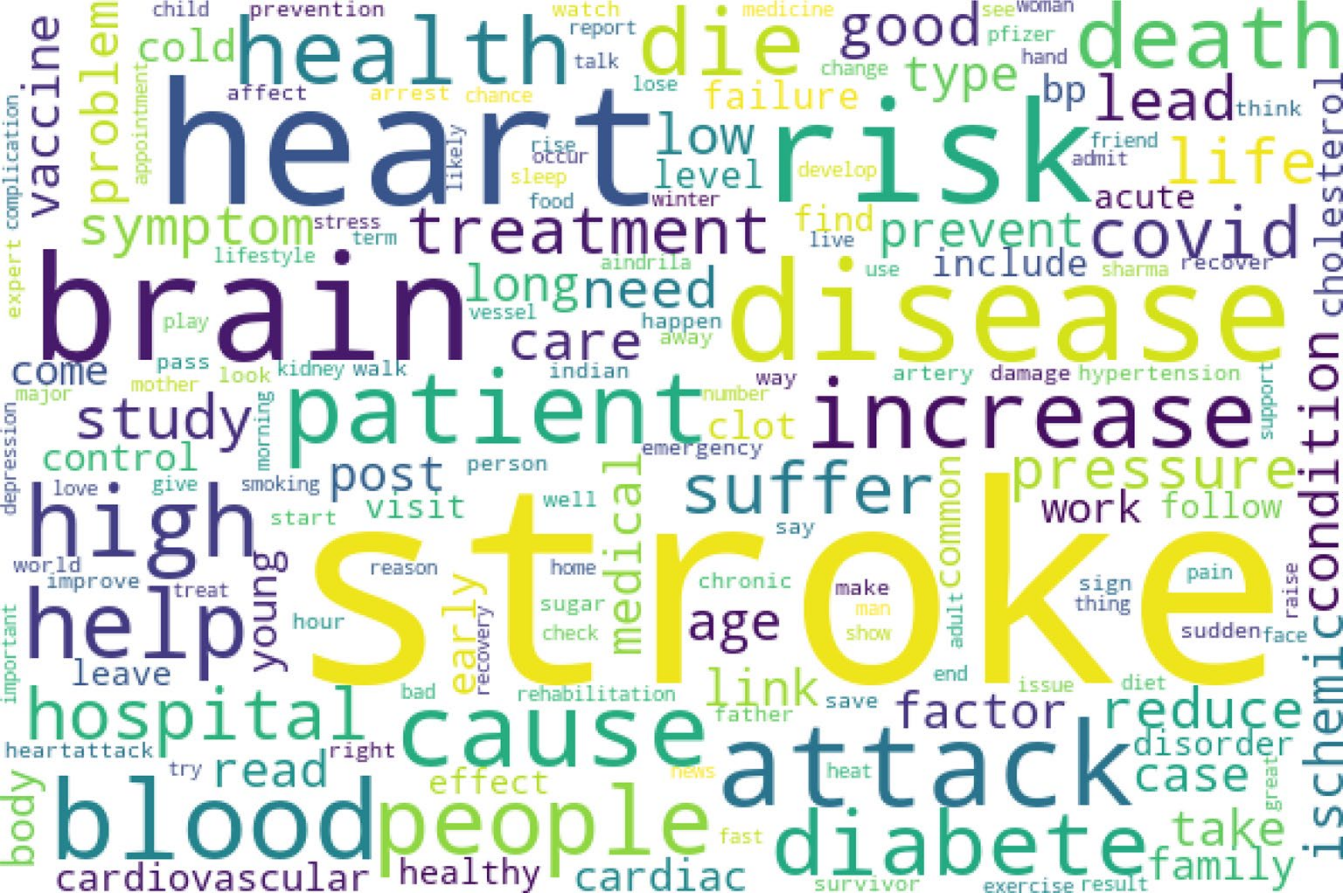
Word cloud of top 150 word tokens from stroke related twitter messages.

The unique stroke tweets (n = 3,561) were analyzed, creating a word cloud from the top 150 tokens (Fig. 1). The top 10 frequently used words were “stroke”, “heart”, “brain” , “risk”, “attack”, “disease”, “blood”, “cause”, “people”, and “patient”. Through a descriptive analysis of word frequencies, we have identified the following key topics of discussion: stroke incidence, COVID-19 vaccine and stroke, rehabilitation, sharing personal experiences, seeking support, research, awareness, information on risk factors, prevention measures, management, and promotion.

**Figure 2:**
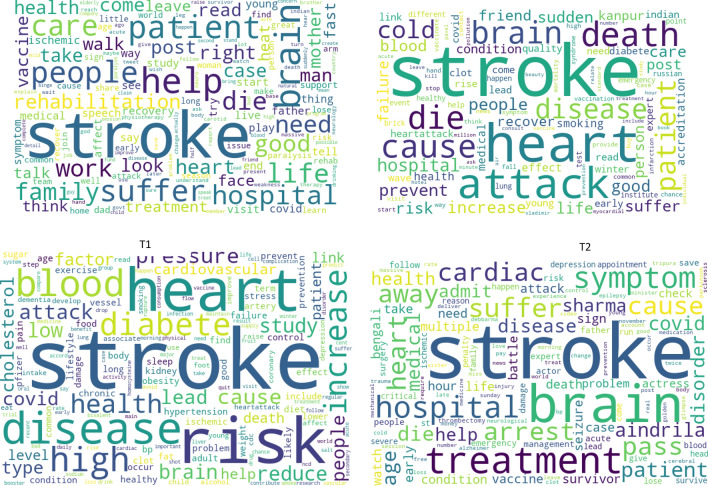
Topicwise word cloud of top 100 words.

Four-topic LDA model generated word clouds to visualize the top 100 word probabilities for each topic. (Fig. 2). Multimedia Appendix 7 reports the beta values for the top 50 words in each topic. Tweet distribution by dominant topic and topic weightage is available in Multimedia Appendix 8. From the analysis, we derived four key topic themes. Table 2 outlines the identified topics, sample tweets, and tweet distribution by user type.

**Table 2:** Identified topics, example tweets, and distribution of tweets by type of user.

Topic	Example tweets	n (%)	Type of user (source) n (%)					
			Dr	HO	Individual (I)	I—Health	Media	Others
Stroke incidence, COVID-19 vaccine and stroke , rehabilitation	My mum just suffered a stroke was in hospital and is recovering slowly now. Terrible thing to happen to a young life. Hope she survives this. Praying!	731 (20.5)	136 (18.6)	65 (8.9)	372 (50.9)	26 (3.6)	120 (16.4)	12 (1.6)
	With persistent and consistent efforts, we have been able to help our patients gain back their independence and mobility. Overcome the effects of post-stroke with us. Contact us today							
	Even my grandfather who had a stroke, and now can't move his entire left side survived the vaccine. Are you sure there isn't any fear mongering going on? My entire family survived the vaccine, extended family included							
Seeking Support, and post-stroke bereavement sharing	I didn't have a single time headache in my entire life but when I had one it was so unbearable it ended in a brain stroke which made me paralyzed	1719 (48.3)	279 (16.2)	238 (13.8)	872 (50.7)	47 (2.7)	237 (13.8)	46 (2.7)
	sir my father got brain stroke we admitted hospital now he is ok in bed doctors told please take home your patient but we don't have a money for discharge almost 45 days spent in hospital please help us sir							
	I used to follow each other on Twitter; interact frequently on DM. He was a veteran soldier who had served in many places in NE. He had shared his mobile no ; we had planned to meet soon. Came to know he passed away today due to stroke							
Risk factors, awareness and prevention	Chronic stress may cause dysregulation of the sympathetic system, endothelial dysfunction, and atherosclerosis. Stress may indirectly increase stroke risk by fostering unhealthy behaviors	520 (14.6)	164 (31.5)	103 (19.8)	150 (28.8)	25 (4.8)	68 (13.1)	10 (1.9)
	An informative Infographic shared by the Centers for Disease control and Prevention on Prediabetes. If you have Prediabetes don't ignore it because it increases your risk for Type 2 diabetes, Heart Disease and Stroke							
	Being physically #active is a crucial step towards good heart #health. It’s one of the most effective ways to strengthen the #heart muscles, warding off artery damage from cholesterol, high #bloodpressure and high blood sugar that can be the reason for #heartattack or #stroke							
Management, research, and promotion	CitiBur Injection is used in the treatment of stroke, head injury, Alzheimer's disease, and dementia in Parkinson's disease. #CitiBur #injection #stroke #headinjury #pcdfranchise #disease #treatment	591 (16.6)	172 (29.1)	127 (21.5)	150 (25.4)	30 (5.1)	101 (17.1)	11 (1.9)
	Conclusion: In this study of 21,655 patients with acute ischemic stroke, it was found that the mean initial in-hospital heart rate was a predictor of all-cause mortality and cardiovascular death, independently of other known risk factors such as age and stroke severity							
	Heart disease refers to a group of conditions that affect the heart and blood vessels, such as coronary artery disease ( #CAD ) , #heartattack, and stroke. If you experience any of these symptoms, it's important to seek medical attention. For appointments call us at XXXXX							
Total		3561 (100.0)	751 (21.1)	533 (15)	1544 (43.4)	128 (3.6)	526 (14.8)	79 (2.2)

*Dr*, Doctor; *HO*, Healthcare organisation; *I*, Individual; Others- Others including non-health organisations.

*Topic 1*: Stroke incidence, COVID-19 vaccine and stroke, and rehabilitation.

The first Twitter topic, comprising 20% of stroke-related messages, discussed stroke occurrences in family, celebrities, and its various aspects. Topics included brain stroke, cold weather-induced stroke, stroke with heart attacks, and recurrent strokes. Users debated COVID-19 and vaccine impacts on stroke—some noted an increase in stroke cases due to weakened immunity, irregular heart rhythms, and blood clots from COVID-19. Others cited positive vaccine experiences. Cancer and co-morbidities' link to stroke, rehabilitation methods (music therapy, physiotherapy, and game-based therapies), and their importance for patient independence and quality of life were also discussed.

*Topic 2*: Seeking support, and post-stroke bereavement sharing.

Twitter's second latent topic centered on seeking support and sharing personal post-stroke experiences, including end-of-life issues in India. This topic dominated the stroke related conversation, accounting for 48.3% of all Indian stroke-related tweets. Users sought various forms of support, like fundraising, job opportunities, medical information, and emotional help. They reached out to celebrities, support groups, doctors, and the public for support. Twitter users shared their coping experiences, physical activities, and determination for a healthy life. Caregivers discussed the challenges faced by stroke patients, including dementia and Alzheimer's, causing stress. Key contributors were individuals (50.7%), doctors (16.2%), and media (13.8%).

*Topic 3*: Risk factors, awareness and prevention.

The latent topic 3 on Twitter centered on stroke risk factors, prevention measures, and awareness messages, comprising 14.6% of all stroke-related tweets. These tweets delved into a spectrum of risk factors, including behavioral aspects such as alcohol consumption, smoking, high salt intake, sleep apnea, stress, and insomnia, metabolic factors like high body mass index, elevated blood sugar levels, high cholesterol, and hypertension, environmental factors such as air pollution and global warming. A key emphasis in these tweets was the intricate interplay between these multiple risk factors and the importance of awareness and the adoption of a holistic, healthy lifestyle encompassing mental and physical well-being.

Furthermore, the public was informed about stroke types, symptoms, early diagnosis, and treatment options. The tweets underscored that stroke is a preventable condition and provided guidance on prevention strategies, including maintaining balanced diet, engaging in regular exercise, and promoting the value of walking and yoga. Notably, doctors and healthcare organizations contributed to approximately half (51.3%) of the tweets within this topic, lending credibility to the conveyed messages.

*Topic 4*: Management, research, and promotion.

This topic constituted 16.6% of the total stroke related messages posted on twitter in India. The importance of initiating the treatment within the golden hour of symptom onset was widely posted. The acronym “FAST” was used to mention the importance of recognition of signs and symptoms. Research studies on burden of stroke, risk factors, treatment methods, drug efficacy, impact of COVID-19, prediction of stroke using machine learning techniques were discussed. Promotional posts showcasing specialist physicians, health facilities, medications, lectures, and workshops were shared. The messages were posted mainly by doctors (29.1%), followed by individuals (25.4%) and healthcare organisations (21.5%).

### Reachability and engagement of stroke messages

Tweet reachability and engagement was assessed using retweet and favorite counts, detailed in Table 3 for each topic and user type. Among the topics, "Seeking support and post-stroke bereavement sharing" had the highest retweet ratio (9.4), followed by "Stroke incidence, COVID-19 vaccine, and stroke, Rehabilitation" (4.1). Regarding favorite ratio, "Seeking support and post-stroke bereavement sharing" had the highest (15.1). The retweet ratio suggests that each "Stroke incidence, COVID-19 vaccine, and stroke" tweet was shared approximately 9.4 times on average. Similarly, the favorite ratio of 15.1 indicates that, on average, around 15 users liked tweets from "Seeking support and post-stroke bereavement sharing." Notably, individuals had a high retweet ratio (9.3), and doctors had a high favorite ratio (19.5), indicating significant reachability.

**Table 3:** Reachability and engagement of stroke related topics and user types on Twitter in India.

Topic/type of user	Number of tweets	Retweet count	Favourite count	Retweet to total tweets ratio	Favourite to total tweets ratio
**Topic**					
Stroke incidence, COVID-19 vaccine and stroke , rehabilitation	731	3023	4945	4.1	6.8
Seeking support, and post-stroke bereavement sharing	1719	16,073	25,966	9.4	15.1
Risk factors, awareness and prevention	520	2039	4712	3.9	9.1
Management, research, and promotion	591	1025	4539	1.7	7.7
**Type of user**					
Doctor	751	5726	14,612	7.6	19.5
Healthcare organisation	533	626	806	1.2	1.5
Individual	1544	14,311	20,572	9.3	13.3
Individual—Health	128	247	605	1.9	4.7
Media	526	666	3005	1.3	5.7
Others	79	584	562	7.4	7.1

### Network analysis

**Figure 3:**
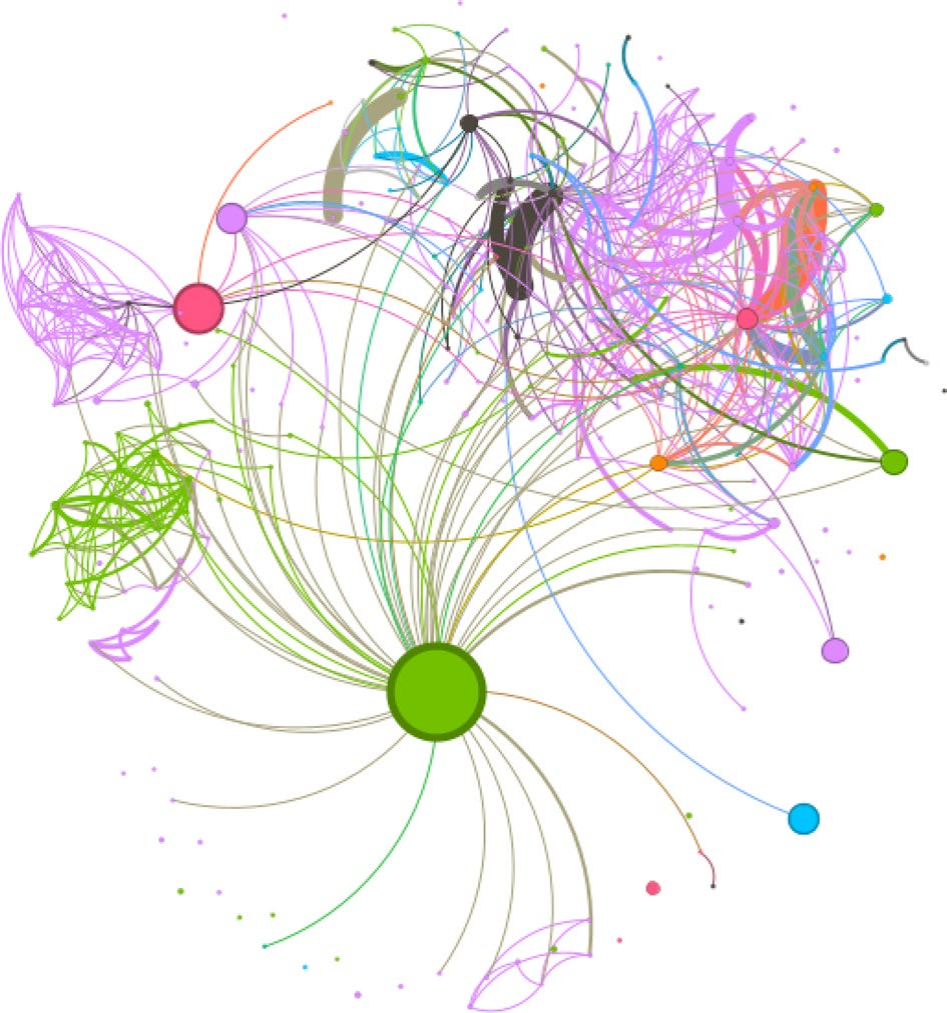
Network of users and influential nodes tweeting about stroke in India.

Figure 3 depicts the network displaying the relationship between the twitter users who conversed regarding stroke in India. Four influential users were identified whose messages has been retweeted or mentioned or quoted more frequently. These relationships indicate the usefulness of messages exhibiting social or intellectual homophily to interact with each other on social media. The network was a sparse network with network density of 0.011, average degree of 4.184, diameter of 9. The influential users were found to be doctors who spread messages on risk factors, research results and occurrence of stroke in a way of spreading awareness to the general public.

## Discussion

The present study examined discussions related to stroke among Twitter users in India from November 2022, to February 2023, spanning a 15-week period. The study sought to comprehend the themes and discourse surrounding stroke, along with characterizing user types and assessing the reach of Twitter messages based on topics and user networks. The study sheds light on what people in India discuss regarding stroke, the most influential stakeholders, and the characteristics of the stroke network in India. Four latent topics were identified, with half of the stroke conversations revolving around seeking support, sharing personal experiences, and discussing end-of-life messages. Also, this topic was found to be the most reachable and conversable among Indian Twitter users. Tweets were originating primarily from individuals, followed by doctors and media. Although individual tweets were more reachable, the tweets from the doctor community were the most conversable. The findings of the study provide evidence that Twitter is a widely utilized platform for sharing and seeking information related to stroke in India, aligning with national and global scientific endeavors^24,25^.

The Altmetric score monitored research popularity on social media, indicating its public reach^26^. Analyzing stroke-related tweets in this study enabled identification of public concerns spanning prevention to end-of-life care. Twitter's accessibility enabled sharing of personal experiences and support-seeking behaviors, aiding coping mechanisms and fostering connections among individuals facing similar challenges. End-of-life messages on social media serve as a means of remembrance and offer comfort to bereaved families^27^.

Similar to studies conducted globally, our study on stroke-related conversations on Twitter in India found that topics such as prevention, stroke recovery, rehabilitation, and raising awareness were commonly discussed. However, topics such as sharing of stroke related resources and Aphasia did not emerge among the Indian Twitter population^27^. It is possible that variations in health-seeking and information-seeking behavior among different populations could account for these differences. Dissemination of accurate and updated information about stroke through social media is important for prevention, awareness, and early diagnosis. Our study found that messages shared by individuals had a higher retweet ratio, consistent with prior studies on non-communicable diseases^13,28–30^. Nevertheless, there's a potential risk of disseminating unauthorized or inaccurate information unintentionally, which could negatively affect those seeking reliable social media information. Conversely, tweets from doctors, known for their credibility and trustworthiness, demonstrated greater influence in terms of the favorite ratio.

The network of users who tweeted stroke messages were sparse, indicating the weak ties. This is the general characteristics of the social media network analysis. The identification of influential nodes inform the stakeholders to communicate more stroke related information to the wider public. Stakeholders, including healthcare providers, government entities, and non-government organizations, must actively supply evidence-based information on the cancer care continuum which will counter the dissemination of misinformation. Implementing a strategic communication plan tailored to the focussed population will improve the chances of effectively reaching the public through social media.

The main advantage of the study is the use of unsupervised LDA topic model that employs traditional natural language processing techniques to identify topics from short text data. This eliminated subjectivity that could arise using a supervised model. However, the findings may not be representative or generalizable to the Indian population, as Twitter users in India are predominantly younger individuals. Although stroke is more prevalent among older adults, promoting strategies such as reducing tobacco and alcohol use, adopting a healthy diet, engaging in physical activity, managing high blood pressure and blood sugar, and maintaining a healthy weight among younger individuals is crucial for stroke prevention, control and strategic planning. Although PROBAST (Prediction model Risk Of Bias Assessment Tool) was not utilised in this study, its significance cannot be overstated in other contexts, as it provides a systematic framework for evaluating bias risk in prediction model studies. Within the scope of this research, PROBAST is pivotal for bolstering the methodological robustness and validity of the study's outcomes. Another important limitation is the exclusion of tweets in Indian languages other than English, which could potentially overlook valuable insights and perspectives from diverse linguistic communities in India.

## Conclusion

Twitter demonstrates promise as a platform for disseminating and sourcing information regarding stroke in India. The topics elucidated in this study provide insight into the discourse within the public sphere, offering valuable data for public health professionals and organizations. This information can inform the development of targeted educational and engagement strategies tailored to specific audience segments. Leveraging social media platforms like Twitter holds potential for augmenting the reach and efficacy of public health initiatives focused on stroke prevention, management, and rehabilitation.

### Supplementary Information

Supplementary Information.

## Data Availability

The dataset used in this study is not publicly accessible due to ethical considerations regarding public tweets. Nonetheless, interested parties can request access to the data from the corresponding author.
