# Supplementary material for: Exploring stroke discourse on Twitter through content and network analysis among Indian users
Source: Sci Rep. 2024 Jul 2;14:15204. doi: 10.1038/s41598-024-65858-9 (PMC11220040; doi:10.1038/s41598-024-65858-9)

Multimedia Appendix 1: Coherence scores for the different number of topics

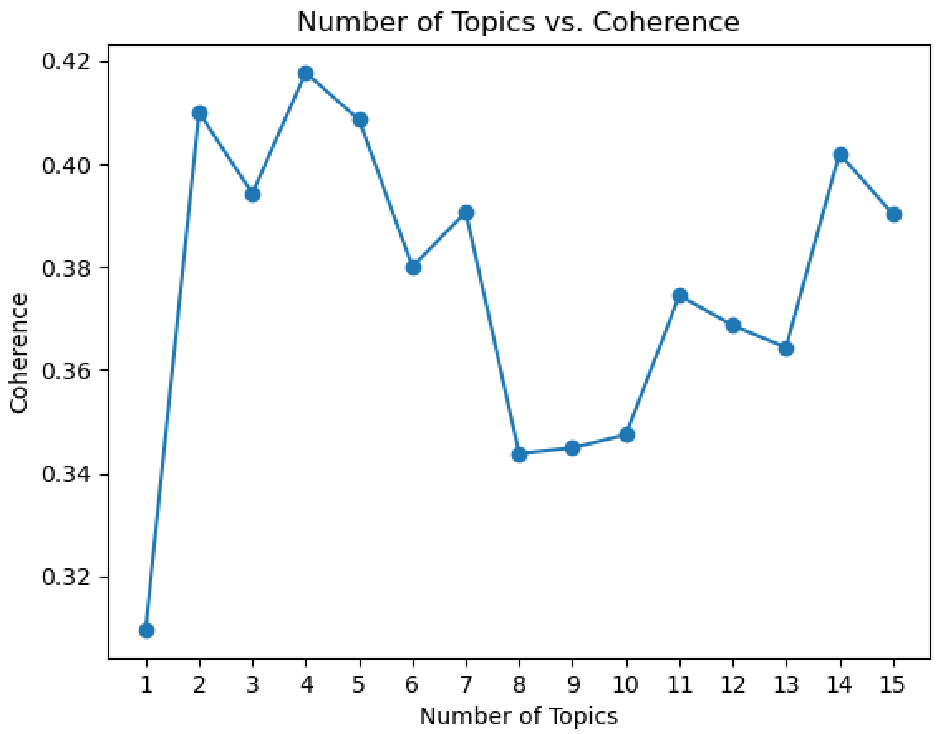

Multimedia Appendix 2: learning decay plot

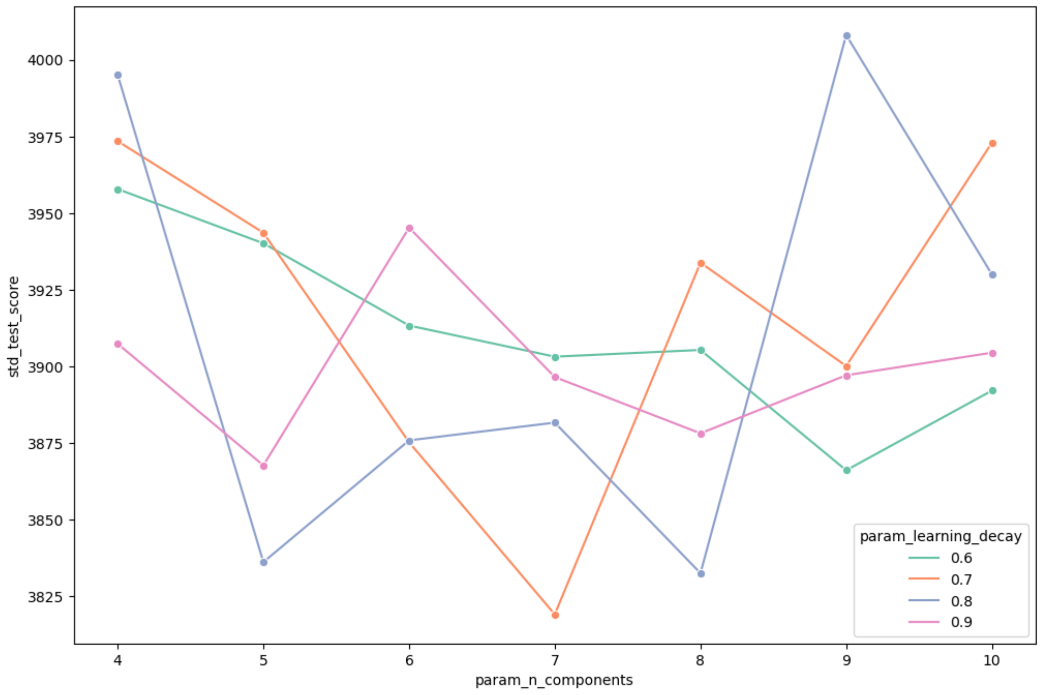

Multimedia Appendix 3: Intertopic distance map

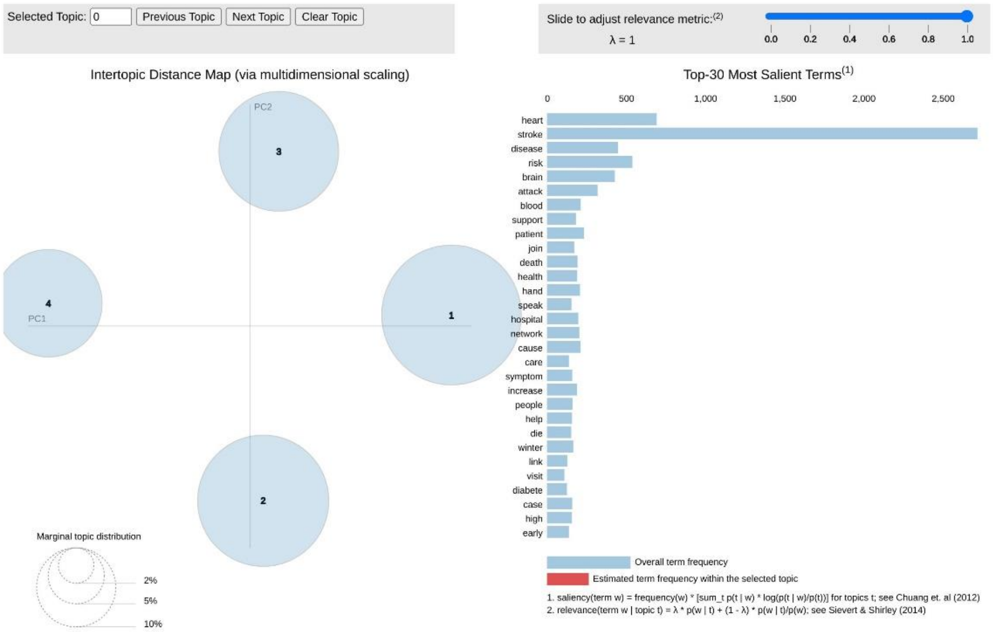

Multimedia Appendix 4: t-distributed stochastic neighbor embedding clustering Visualized

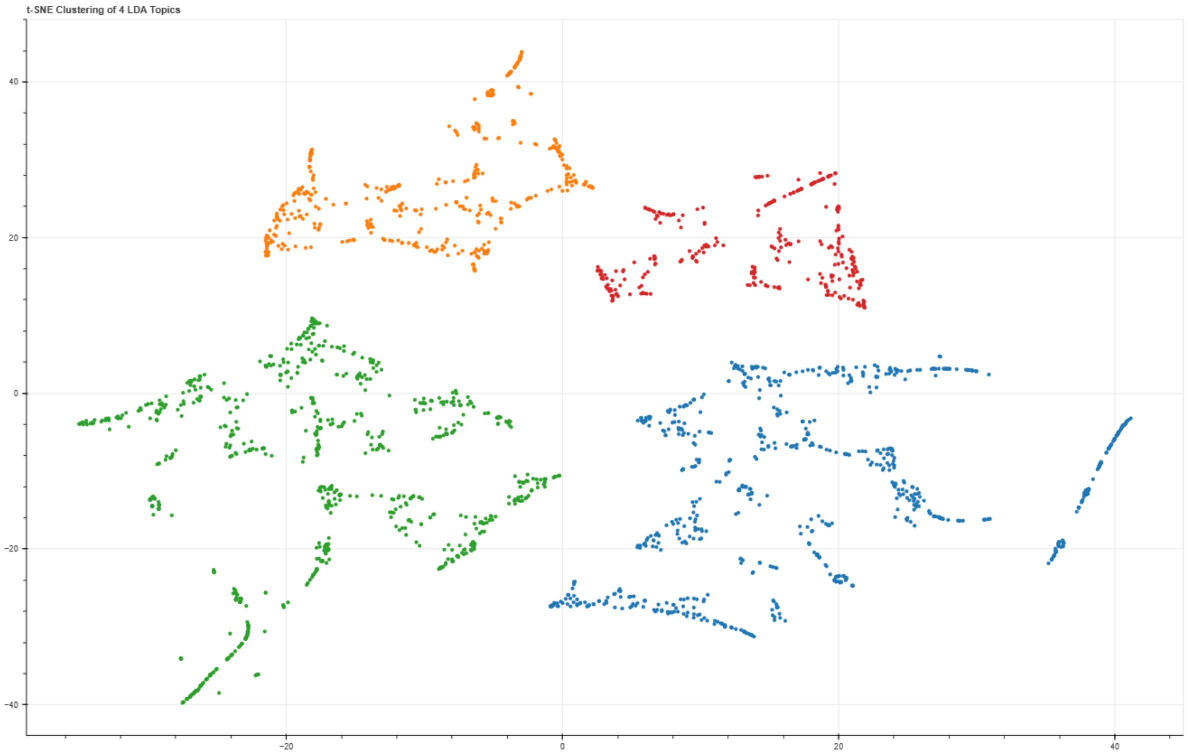

Multimedia Appendix 5: Frequency of stroke related tweets during the study period (7th November 2022 – 28th February 2023)

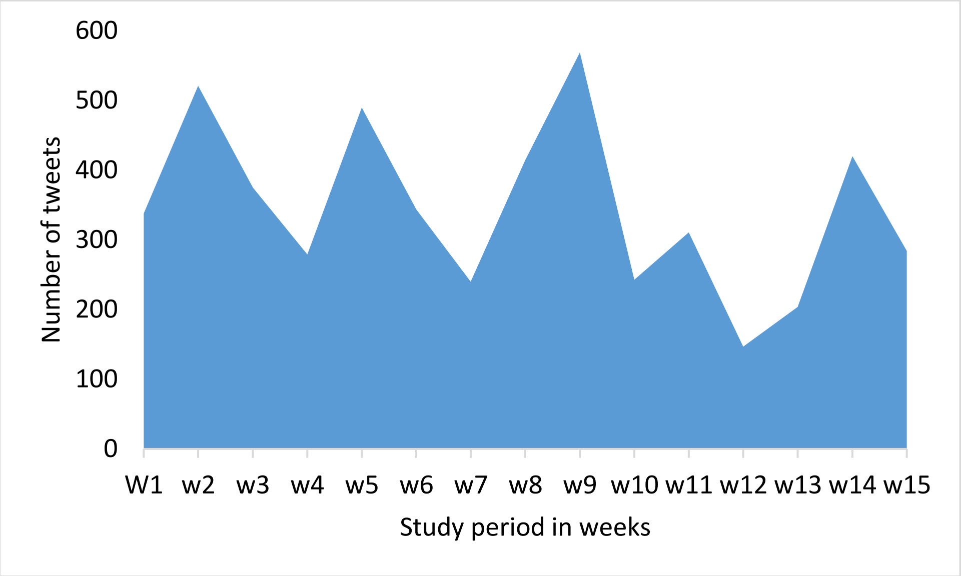

Multimedia Appendix 6: Distribution (%) of type of user tweeted regarding stroke during the study period

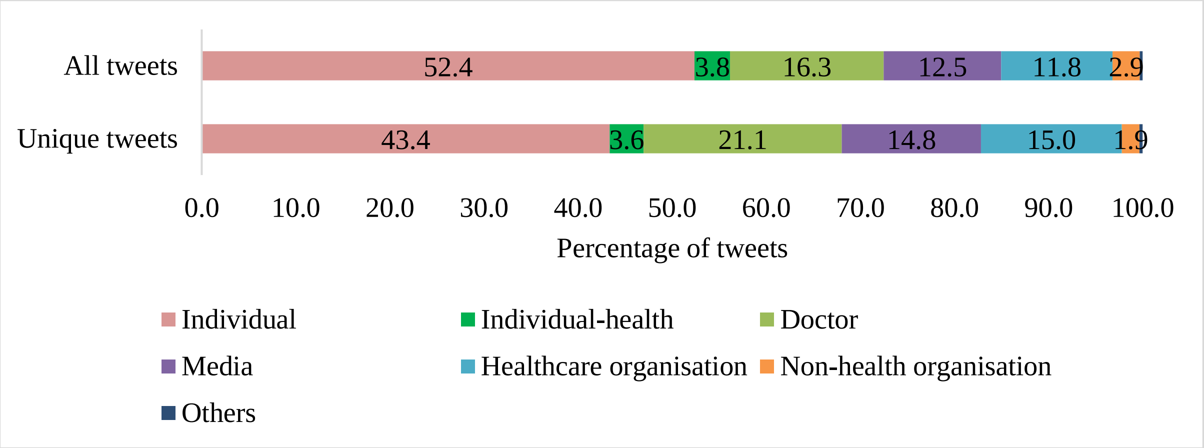

Multimedia Appendix 7: List of top 50 probability (beta) distributions of words in each topic.

No of words	Topic 1		Topic 2		Topic 3		Topic 4	
	Word	Beta	Word	Beta	Word	Beta	Word	Beta
1	stroke	0.12811720	stroke	0.0559709	stroke	0.07566495	blood	0.0304228
2	brain	0.04101411	disease	0.0518070	heart	0.05857105	support	0.0261018
3	patient	0.02247303	death	0.0222433	risk	0.04560249	join	0.0246290
4	hand	0.01994238	health	0.0219823	attack	0.02705113	speak	0.0220927
5	network	0.01948777	symptom	0.0183926	cause	0.01788945	care	0.0198990
6	hospital	0.01895024	link	0.0148901	increase	0.01606583	visit	0.0156366
7	people	0.01535499	diabete	0.0144575	winter	0.01404340	hub	0.0135939
8	help	0.01516048	diabetes	0.0127328	case	0.01353213	suffer	0.0116870
9	die	0.01475953	launch	0.0118361	high	0.01334523	zydushospital	0.0108712
10	early	0.01324025	develop	0.0104796	type	0.01064205	think	0.0103541
11	need	0.01290866	level	0.0100858	lead	0.00896619	appointment	0.0101810
12	treatment	0.01056477	common	0.0087422	reduce	0.00864135	hold	0.0090798
13	take	0.00896252	prevent	0.0074427	condition	0.00840770	try	0.0085989
14	artery	0.00861617	make	0.0071533	control	0.00789000	damage	0.0083526
15	good	0.00850781	show	0.0067028	rise	0.00788943	work	0.0083415
16	follow	0.00823247	little	0.0062716	come	0.00745653	head	0.0072172
17	zydushospital	0.00699663	important	0.0061933	study	0.00733936	clot	0.0070630
18	improve	0.00618097	depression	0.0060279	effect	0.00715611	youngster	0.0070475
19	relate	0.00596677	base	0.0058581	predict	0.00705336	live	0.0063144
20	rehabilitation	0.00563698	carry	0.0057309	heartattack	0.00702682	way	0.0060165
21	save	0.00562804	premature	0.0056145	read	0.00667455	person	0.0054645
22	day	0.00537795	well	0.0054601	world	0.00662348	record	0.0054471
23	second	0.00532702	thank	0.0052117	life	0.00658979	go	0.0054114
24	change	0.00506575	affect	0.0051801	problem	0.00644346	block	0.0052747
25	recover	0.00505710	include	0.0050318	cent	0.00627287	fall	0.0047059
26	post	0.00478000	prevention	0.0050056	age	0.00623949	session	0.0046138

27	home	0.00472925	hour	0.0049871	cholesterol	0.00620850	mention	0.0045986
28	recovery	0.00468120	stress	0.0049376	sign	0.00593360	buy	0.0045758
29	hub	0.00467234	look	0.0048309	covid	0.00591314	contact	0.0044747
30	consult	0.00440602	give	0.0047335	blood_vessel	0.00576333	medical	0.0044403
31	close	0.00421195	major	0.0046310	pain	0.00549759	woman	0.0042711
32	speech	0.00414693	able	0.0045140	expert	0.00546675	tell	0.0042168
33	therapy	0.00411043	hypertension	0.0044857	body	0.00537391	talk	0.0041914
34	surgery	0.00408986	system	0.0044088	occur	0.00528935	leave	0.0038887
35	critical	0.00407489	alzheimer	0.0043284	factor	0.00512370	soon	0.0038521
36	doctor	0.00405406	great	0.0041448	find	0.00491695	right	0.0037278
37	morning	0.00381621	get	0.0040918	walk	0.00474956	break	0.0037248
38	detail	0.00374627	cell	0.0038896	low	0.00467659	officer	0.0036679
39	possible	0.00358026	liver	0.0038079	blood_pressure	0.00447583	yesterday	0.0036219
40	tripura	0.00352786	remain	0.0037884	young	0.00444548	team	0.0035967
41	survivor	0.00351070	outcome	0.0037712	treat	0.00443759	child	0.0035761
42	information	0.00343521	obesity	0.0037583	report	0.00409593	wish	0.0035136
43	senior	0.00340859	daily	0.0036231	cardiovascular	0.00399944	single	0.0035011
44	situation	0.00323360	severe	0.0035285	say	0.00398123	illness	0.0034712
45	fast	0.00323269	forget	0.0035268	simple	0.00390063	history	0.0034711
46	paralysis	0.00315270	poor	0.0035179	uncontrolled	0.00388958	touch	0.0034131
47	face	0.00310776	experience	0.0035102	issue	0.00386283	late	0.0033716
48	check	0.00306587	parkinson	0.0034964	food	0.00384902	breath	0.0031773
49	action	0.00301987	drug	0.0034646	chance	0.00384259	vascular	0.0031747
50	start	0.00292776	require	0.0034039	healthy	0.00381335	physician	0.0031032

Multimedia Appendix 8: Distribution of tweets by dominant topic and topic weightage

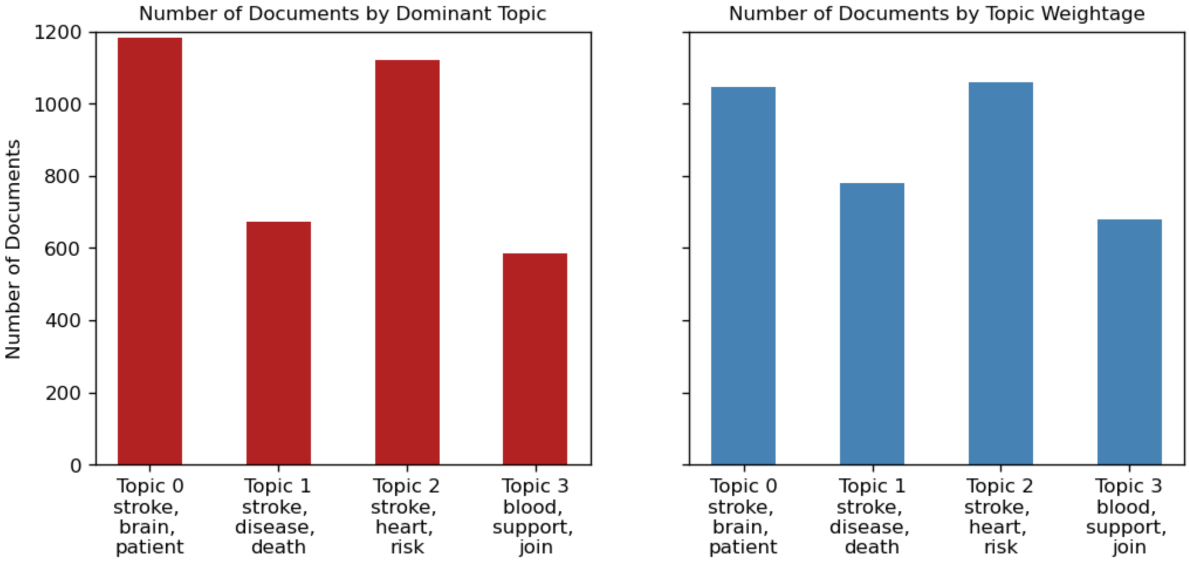

Supplement: Supplementary file 1 — Supplementary Information. [file 41598_2024_65858_MOESM1_ESM.pdf]
